# Flare or foe? - *Mycobacterium marinum* infection mimicking rheumatoid arthritis tenosynovitis: case report and literature review

**DOI:** 10.1186/s41927-020-0114-3

**Published:** 2020-03-16

**Authors:** Nils Schubert, Tillmann Schill, Marlene Plüß, Peter Korsten

**Affiliations:** 1 Department of Nephrology and Rheumatology, University Medical Center Göttingen, Robert-Koch-Str. 40, D-37075 Göttingen, Germany; 2 Department of Dermatology, Venereology, and Allergology, University Medical Center Göttingen, Göttingen, Germany

**Keywords:** Rheumatoid arthritis, *Mycobacterium marinum*, Immunosuppressive agents, Atypical mycobacteria

## Abstract

**Background:**

Rheumatoid arthritis is the most common type of inflammatory arthritis affecting about 1% of the population. With the advent of disease-modifying anti-rheumatic drugs the disease can be well controlled in many cases. Patients, however, are prone to developing infectious complications. In rare cases, these can mimic a flare of the underlying itself.

**Case presentation:**

We report the case of a 45-year-old female patient with a history of seronegative rheumatoid arthritis (RA) who presented with swelling and tenderness of the third metacarpophalangeal joint of the right hand. A flare of her RA was suspected based on clinical and ultrasound findings which showed a tenosynovitis with intense power doppler activity. Her steroid dose was increased but the clinical response to glucocorticoid therapy was very limited. Subsequently, she developed skin manifestations of ‘swimmer’s granuloma’ over the next 2 weeks after first presentation. Finally, a diagnosis of a *Mycobacterium marinum* infection was established with the help of tissue biopsy and culture, and the patient received appropriate antibiotic treatment with the desired effect.

**Conclusions:**

This case highlights the difficulty of distinction between infection and inflammation in patients with joint swelling and pain, especially in the age of disease-modifying drugs (DMARDs) and the concomitant risk of atypical infections. A review of the literature identified eight additional published cases, which suggests that *Mycobacterium marinum* infection is a rare but recognized complication of DMARD therapy. It can mimic a flare of the underlying arthritis potentially leading to diagnostic delays, and requires differential diagnostic methods to identify the pathogen and pave the way for appropriate treatment.

## Background

Rheumatoid arthritis (RA) is the most common type of inflammatory arthritis worldwide with a prevalence of about 1% [1]. Over the past 20 years, the development and use of different immunosuppressive disease-modifying antirheumatic drugs (DMARDs) has been steadily increasing [2]. With the advent of biosimilars (bsDMARDs) and the subsequent cost reduction, their use is likely to increase further. While this has clearly been beneficial for many patients, side effects, such as infections, occur more frequently [3]. The use of immunosuppressants, particularly tumor necrosis factor-alpha inhibitors (TNF-i), can render patients susceptible to infections with *Mycobacterium tuberculosis (MT)* or to a reactivation of a latent tuberculosis [4]. In addition to MT, there is a large number of *nontuberculous mycobacteria (NTM),* which represent the majority of mycobacteria [5]. They can infect humans as well as animals, as they inhabit a variety of similar surroundings, such as drinking water or natural waters [6, 7]. A particular risk factor for acquiring an infection with NTM is the frequent use of immunosuppressants [8].

Among the NTM, one species is *Mycobacterium marinum (MM)*. This bacterium, occurring in both salt and fresh water, grows optimally at a temperature between 30 and 32 °C [9] and, in case of an infection, shows an incubation time of typically two to 4 weeks [10]. It can lead to diseases in various fish species and subsequently to infections in humans who come into contact with infected fish or contaminated water, for example during the cleaning process of aquaria by direct inoculation into previously injured tissue [10, 11]. Usually, superficial soft tissue infections occur with the formation of red nodules, ulcerations and abscesses [12–14]. In some cases, however, tenosynovitis, arthritis and osteomyelitis may occur [15, 16].

Here, we present the case of a female patient with known RA treated with immunosuppressive therapy who presented with pain of her right hand secondary to an infection with *MM*. In addition, we reviewed the literature for similar cases in order to provide clinicians with an overview on the topic.

## Case presentation

The 45-year-old female patient had received a diagnosis of seronegative RA 4 years before presentation. She had received several DMARDs including methotrexate (MTX), sulfasalazine (SSZ), and hydroxychloroquine (HCQ). Her current treatment included leflunomide (LEF) and intermittent usage of low-dose prednisolone. Her past medical history was significant for arterial hypertension, which was treated with metoprolol and amlodipine.

**Fig. 1:**
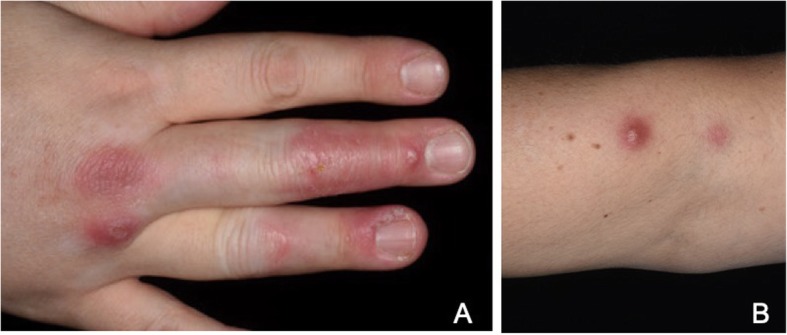
Clinical images of the patient (**a**) Reddish-purplish discoloration on the back of the distal and intermediate phalanges of the third and fourth finger as well as the third and fourth metacarpophalangeal joint. On the base of the erythema, there are several formed papules and nodules with a central golden scab. **b** Reddish-purplish nodule on the proximal forearm

**Fig. 2:**
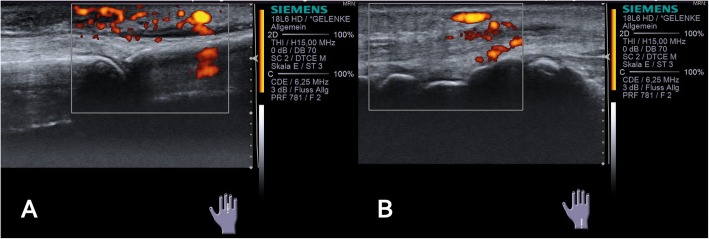
Ultrasound images of the third metacarpophalangeal joint. **a** Longitudinal view of the third metacarpophalangeal joint showing intense power doppler signaling III°. **b** Longitudinal view of the carpal bones demonstrating tenosynovitis of the fourth extensor tendon sheath with power doppler signaling III°

The patient presented with acute symptoms, as she had been suffering from increasing pain and swelling of her right hand. Two weeks before presentation, she had already taken 20 mg prednisolone for 10 days with only slight improvement of symptoms. On presentation, there was swelling and tenderness of the third metacarpophalangeal (MCP) joint of the right hand. A flare of her RA was assumed and prednisone increased to 40 mg daily. Another 2 weeks later, she returned for a follow-up visit with new cutaneous findings: Clinical examination at that time revealed a visible and palpable swelling of the back of the right hand and third MCP joint (Fig. 1a). Other joints were not affected. A musculoskeletal ultrasound was performed and showed tenosynovitis of the third extensor tendon with increased power doppler signal (Fig. 2a and b). Laboratory analysis showed a thrombocytosis of 422 × 10^3/μl (normal range, NR: 150–350 × 10^3/μl), a leukocytosis of 15,9 × 10^3/μl (NR: 4–11 × 10^3/μl) and slightly increased CRP of 7,6 mg/l (NR: ≤ 5 mg/l). The differential blood count showed increased neutrophilic granulocytes with 83,9% (NR: 40–76%). Otherwise, there were no pathological findings.

Due to the novel skin findings, the patient was urgently referred to our institution’s Dermatology Clinic. In addition to the findings on the right hand, a complete examination of the skin revealed a single reddish-purplish erythematous nodule in a linear “sporotrichoid” form on the right proximal forearm (Fig. 1b). No palpable lymph nodes were found in the axillary region. Upon further questioning, the patient reported regular cleaning of fish tanks and recent travel to the seaside. A clinical diagnosis of a so-called “swimmer’s granuloma” was made and a biopsy scheduled for microbiological analysis. In light of this information, the nailfolds of the third and fourth right finger seemed to be the most likely site of inoculation. The histopathological analysis revealed granulomatous inflammation with necrosis. Ziehl-Neelsen staining and blood-based testing for *Mycobacterium tuberculosis* (using Quantiferon TB Gold Plus® test) were negative. After several weeks, infection with *MM* was confirmed in the tissue culture.

### Treatment and outcome

Based on the clinical findings, immediate antibiotic treatment with clarithromycin and rifampicin was initiated. There was gradual improvement of the clinical findings over the following months.

## Discussion and conclusions

In the present case, the patient presented with swelling of one joint and sonographic examination confirmed intense inflammation. Thus, both a flare of her RA and infectious complications needed to be considered. This differentiation is notoriously difficult [17–19]. Of note, the skin findings had not been present during the first clinic visit. Therefore, an important step in the management of this patient was a close follow-up and appropriate referral to Dermatology after the appearance of new skin findings, where the correct diagnosis was made.

The appearance of satellite lesions proximal to the entry site, as in our case, is likely due to lymphatic spread and critical for the diagnosis: This type of spread has been coined as “sporotrichoid”, as it resembles a fungal infection with *Sporothrix schenkii*. There are various clinical variants described with this latter agent, the one closest resembling an infection with *MM* is the “lymphocutaneous” phenotype [20, 21]. Very few agents appear in a similar pattern: These include *MM*, Sporothrix schenkii, nocardiosis, leishmaniasis, tularemia, and other bacterial or fungal infections [21].

The diagnosis of a *MM* infection can be achieved with typical clinical findings of a “swimmer’s granuloma” and confirmation of the pathogen in tissue culture. It should be noted that the biopsy must be cultured at a temperature between 30 and 32 °C for a period of 6 weeks. A positive culture can be obtained in about 70–80% of cases [10]. The therapy consists of a combined antibiotic therapy of either ethambutol or clarithromycin with rifampicin, and potentially also surgical debridement [22].

**Fig. 3:**
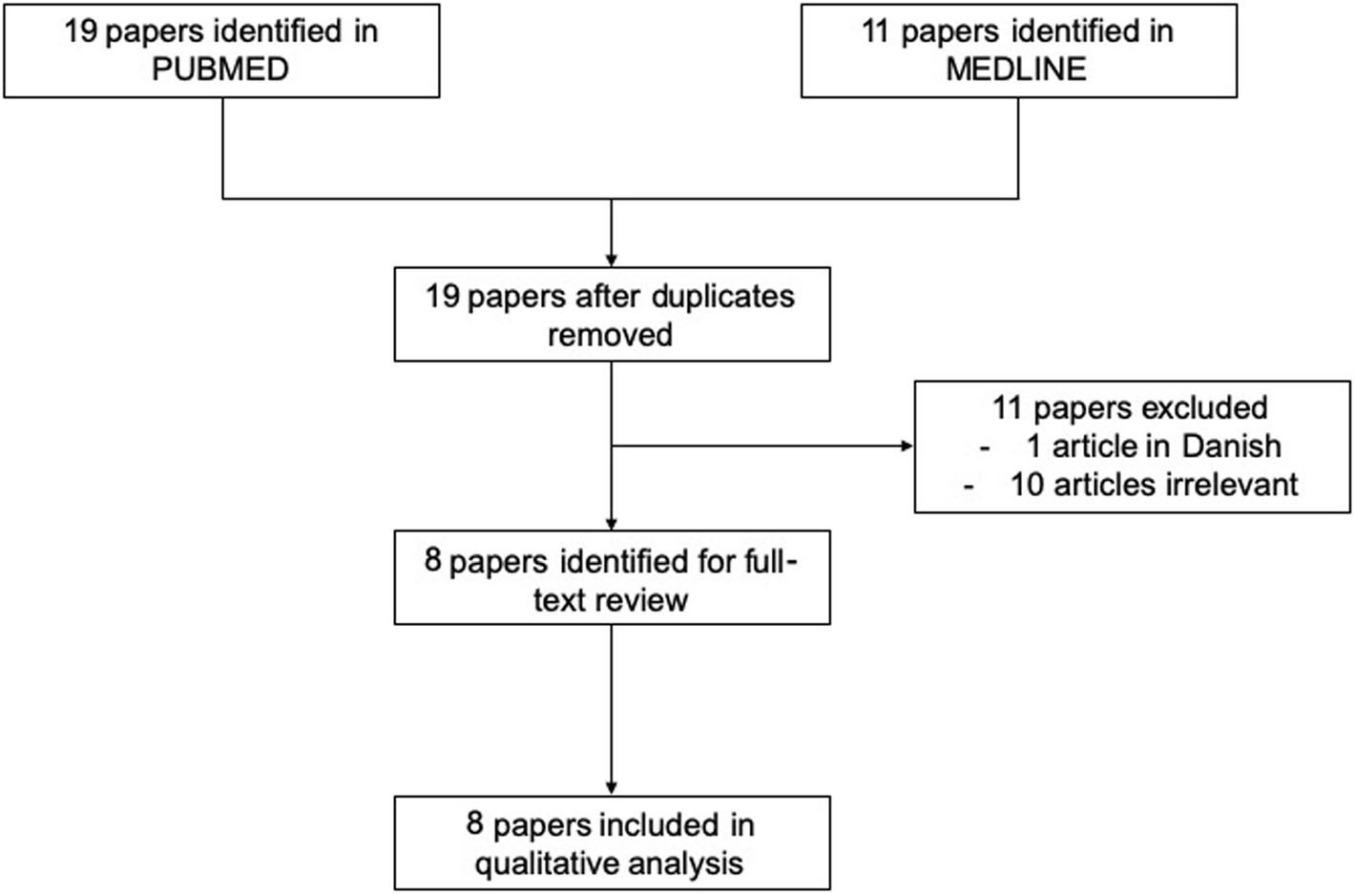
Flow chart of identified articles

We searched PubMed and MEDLINE (using the search terms “*Mycobacterium marinum*” AND “rheumatoid arthritis“) for cases where *MM* infection occurred secondary to immunosuppressive therapy or mimicked RA at first presentation. 19 publications were identified. After review of these, eleven were excluded (Fig. 3). We further analyzed the remaining eight cases and compared them qualitatively with our case (Table 1).

**Table 1:** Detailed review of published cases

First author, year	Timing of onset of infection	Clinical symptoms	Immunosuppressive agents used	Treatment	Outcome
Böcher, 2002	Not described	livid nodules on hands, forearms, and feet, necrotizing skin ulcers, tendon rupture; fever	MTX	CPF, ETA, CLM	Clinical remission
Chopra, 2003	2 years after RA-diagnosis	wrist swelling of the right hand	ETN	CLM	Clinical remission
Roddy, 2008	5 years after RA-diagnosis	erythematous macules and papules. Following progression to pustules and fluctuant nodules, which both hands and arms	MTX	CPF, CLM,, DXC	still recovering
Hess, 2009	Not described	right fourth digit was swollen and associated with fissuring and crusting; subcutaneous nodules on dorsal hand and forearm	MTX, SSZ, INF	AZM, ETA, TMT	improvement but still significant functional impairment of the finger
Danko, 2009	15 years after RA-diagnosis	erythematous nodules on upper thighs and lower lip	MTX, INF	RAP, INH, PZA, ETA	No new lesions
Caron, 2011	Treatment over the previous 18 months	Inflammatory lesion on her right index and nodules on the ipsilateral forearm	ADA	CLM, MC	Clinical remission
Bakker, 2913	Not described	Erythematous livid papules and necrotic nodules on right hand and both legs	MTX, ADA	ETA, CLM	Clinical remission
Papathemell, 2016	Not described	subcutaneous nodules on her legs	LEF, AZA	ETA, CLM, RAP	Stabilization of the disease

*ADA* adalimumab, *AZA* azathioprine, *AZM* azithromycine, *CPF* ciprofloxacine, *CLM* clarithromycine, *DXC* doxycycline, *ETA* ethambutol, *ETN* etanercept, *INF* infliximab, *INH* isoniazid, *LEF* leflunomide, *MTX* methotrexate, *MC* minocycline, *PZA* pyrazinamide, *RFB* rifabutin, *RAP* rifampicine, *SSZ* sulfasalazine, *TMT* trimethaprim

**Fig. 4:**
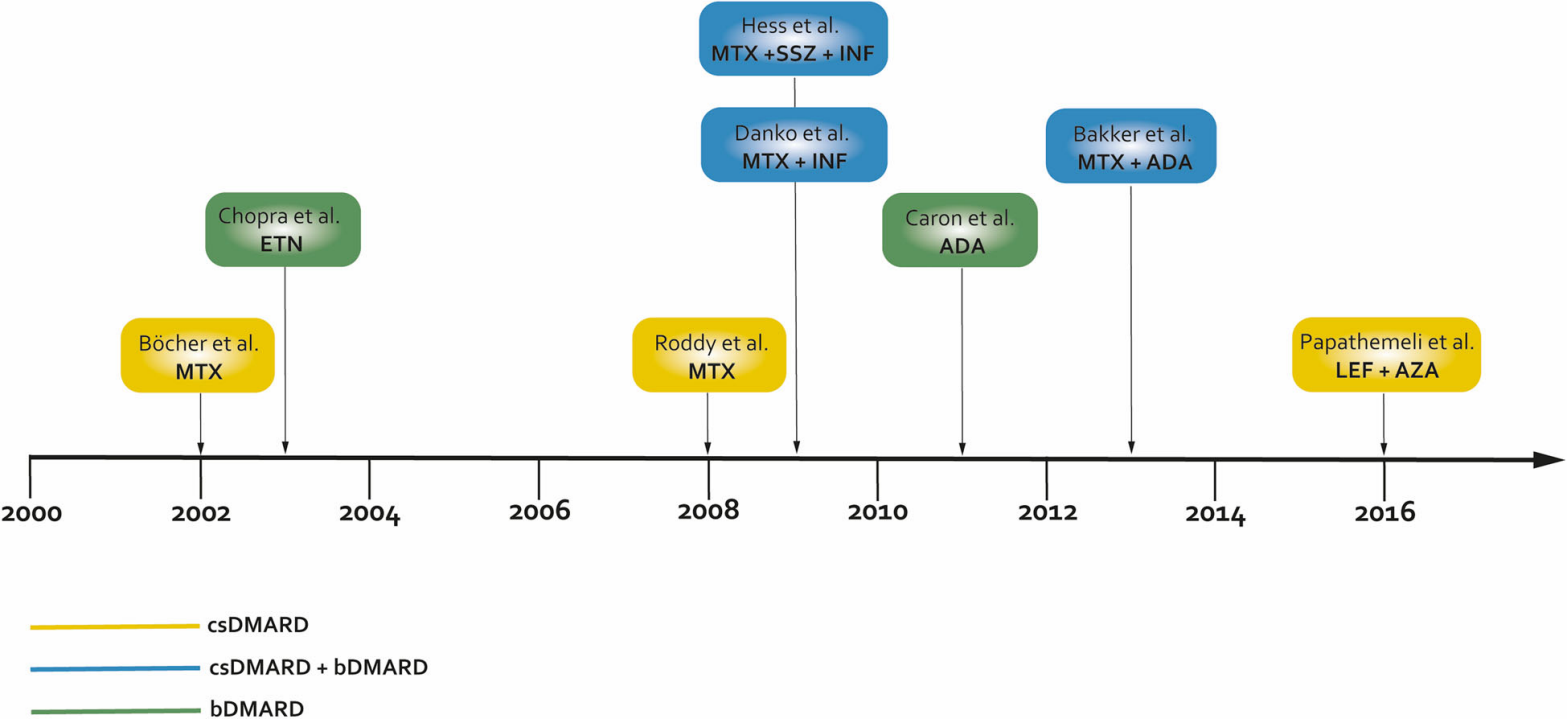
Overview of published cases in relation to treatment with conventional or biological disease-modifying anti-rheumatic drugs. ADA, adalimumab; AZA, azathioprine; b, biological; cs, conventional synthetic; DMARD, disease-modifying anti-rheumatic drug; ETN, etanercept; INF, infliximab; LEF, leflunomide, MTX, methotrexate, SSZ, sulfasalazine

In the case presented within this report, the patient received a single cDMARD therapy with LEF. In two of the eight cases, there was also a single conventional DMARD. Both of these patients received MTX [23, 24]. In two other cases, there was a single therapy with a bDMARD. The patients received Etanercept and Adalimumab [25, 26]. In the majority of published cases, a combination therapy with a cDMARD and a bDMARD was present [27–29]. In one published case a combination of two cDMARDs and one bDMARD was described [30]. In five of the included cases the patients received either single therapy with a TNF-i or a combination therapy of a TNF-i with another drug [25–28, 30]. Figure 4 summarizes the published cases.

In more than half of the published cases, a TNF-i was included in the therapeutic regimen. It is known from several studies that TNF-i increase the risk of reactivation of a latent tuberculosis and new infection with tuberculosis [4]. Therefore, testing for concomitant or dormant MT infection before initiation of therapy with these agents is considered standard of care. Nevertheless, despite this testing, infections with NTM may occur.

However, other predisposing factors also seem to exist: It may be sufficient to receive a single immunosuppressive agent in the presence of a superficial injury to promote infections with *MM*. Based on our literature review, no single agent with an exceptionally high risk could be identified. Of note, however, agents with a different mechanism of action, such as co-stimulation blockers (abatacept), anti-interleukin 6-blockers (tocilizumab, sarilumab), b-cell depleting agents (rituximab) or janus kinase inhibitors (baricitinib, tofacitinib) have not been associated with *MM* infections.

In summary, the occurrence of *MM* infection in the presence of DMARD therapy is very rare. One explanation is that it is generally a very rare infection, even in the immunocompetent population. Another possible explanation is that these infections are often misjudged clinically as a flare of existing RA. Table 2 summarizes potential characteristics which may differentiate an *MM* infection from a RA flare.

**Table 2:** Characteristics differentiating *Mycobacterium marinum* infection from rheumatoid arthritis flare

	Favoring MM infection	Favoring RA flare
Arthritis	Rare	Frequent
Tenosynovitis	Rare	Frequent
Skin findings	Typical swimmer’s granuloma with reddish-purplish discolouration	Rare presence of rheumatoid vasculitis or pyoderma gangraenosum
Laboratory findings	Elevated inflammatory markers (White blood count, C-reactive protein)	Leukocytosis rare C-reactive protein in severe flares
Response to glucocorticoid therapy	No improvement	Usually improves

*MM Mycobacterium marinum*, *RA* rheumatoid arthritis

The reported case highlights the importance of clinical judgement in the assessment and follow-up of RA patients treated with DMARDs, since complications may arise and can be mistaken for a flare of the underlying disease. Therefore, further diagnostic interventions including biopsy and microbiological studies may be required, especially in patients not responding as expected to immunosuppressive agents. The most important clinical clue in this case was the appearance of new skin lesions which, ultimately, led to the correct diagnosis.

## Data Availability

All data are contained within the manuscript.

## Abbreviations

CRP: C-reactive protein
DMARD: Disease-modifying anti-rheumatic drugs
HCQ: Hydroxychloroquine
LEF: Leflunomide
MCP: Metacarpophalangeal
MM: *Mycobacterium marinum*
MT: *Mycobacterium tuberculosis*
MTX: Methotrexate
NTM: Non-tuberculous mycobacteria
RA: Rheumatoid arthritis
SSZ: Sulfasalazine
TNF-i: TNF-alpha inhibitor
